# Safety of Total Knee Arthroplasty in the Treatment of Knee Osteoarthritis and Its Effect on Postoperative Pain and Quality of Life of Patients

**DOI:** 10.1155/2021/6951578

**Published:** 2021-12-22

**Authors:** Yunfeng Zhang, Hua Liu

**Affiliations:** Department of Joint Surgery, Ningbo No. 6 Hospital, Ningbo 315100, Zhejiang, China

## Abstract

**Objective:**

To explore the safety of total knee arthroplasty (TKA) in the treatment of knee osteoarthritis (KOA) and its impact on patients' postoperative pain and quality of life.

**Methods:**

A total of 60 KOA patients admitted to our hospital from January 2019 to January 2020 were selected as the research objects. The knee joint scores (HSS) before and after TKA were compared, and the patients' quality of life was evaluated using the Osteoarthritis Index of Western Ontario and McMaster University (WOMAC). At the same time, the number of patients with complications was recorded, and the efficacy of TKA was comprehensively analyzed.

**Results:**

The postoperative HSS score was significantly higher than the preoperative score (*P* < 0.05), the postoperative pain score increased with time, and the pain gradually decreased. The postoperative WOMAC score was significantly lower than the preoperative score (*P* < 0.001), and the score at 6 months after surgery was significantly lower than that at 3 months after surgery (*P* < 0.001). There were no complications such as severe prosthesis fracture, secondary sepsis, and patellar tendon rupture, and the total incidence of complications was 11.7%. The effective rate of treatment at 6 months after operation was 98.3%, which was significantly higher than that at 3 months after operation (*P* < 0.05).

**Conclusion:**

Total knee arthroplasty can improve the knee joint function of patients with knee osteoarthritis, with low postoperative pain, low complication rate, and good quality of life for patients. It is worthy of promotion and application.

## 1. Introduction

In recent years, more people suffer from knee osteoarthritis (KOA) all around the world with the growing population of old people, and this disease will lead to physiologic dysfunction in knee joints. The patients not only suffer from pain and swelling of joints but also face the risk of completely losing the knee function, so the interventional therapy should be carried out as soon as possible to ensure their life and health [1–3]. At present, oral analgesic medication and other drug therapies are the key methods to treat patients with mild KOA, but drug therapies cannot fully meet the needs of knee joint rehabilitation of patients with severe KOA, so surgery is an important measure to protect the knee function of patients with severe KOA. The most common surgical treatments include total knee arthroplasty (TKA) and arthroscopic debridement [4–7]. TKA is a comprehensive expression of medical science and can be used for treating various terminal stage diseases of knee joints. In recent years, the continuous optimization of related technologies improves the curative effect of TKA, and practical achievements show that TKA has a positive effect on relieving pain and promoting the recovery process of knee function of patients. Based on this, this study aims to explore the actual application effect of TKA, and 60 cases with KOA admitted to our hospital from January 2019 to January 2020 were selected for the study, with summary as follows.

## 2. Materials and Methods

### 2.1. General Data

60 cases with KOA admitted to our hospital from January 2019 to January 2020 were selected as the research objects, with the general data of patients given in Table 1. This study was approved by the hospital ethics committee.

### 2.2. Inclusion Criteria

The inclusion criteria for the patients of this study were as follows: the patients or their family members were fully aware of the research process and signed the informed consent, the patients were diagnosed with KOA [8–11] by examination and had knee pain and other symptoms, the patients were treated by TKA, and the patients who were older than 45 years old.

### 2.3. Exclusion Criteria

The exclusion criteria for the patients of this study were as follows: the patients with mental problems or who were unable to communicate with others, the patients who were suffering from other organic diseases, the patients who were not available for the follow-up, the patients with knee joint infection and other diseases, the patients with immune dysfunction, and the patients with old fracture of lower extremity.

### 2.4. Methods

All patients underwent the treatment of TKA, with the specific procedures as follows. (1) The patients were given routine preoperative examination, were evaluated for their loading ability of lower limbs, were given dietary intervention, and were informed of the TKA surgery details, which would make the patients psychologically prepared. (2) The supine position was taken after the combined spinal-epidural anesthesia, and the disinfectant towel was draped under the affected limb after cleaning and disinfection of the affected limb, and then, tourniquet was used after blood evacuation. (3) The vertical incision was made at the anterior of the affected knee joint, and the patients' skin, fascia, and joint cavity were incised successively. Then, the patella was opened to remove the proliferative synovial tissue, resect medial and lateral meniscus, and resect anterior cruciate ligament. (4) The distal standard osteotomy was performed after the distal femoral osteotomy locator was installed, and then, tibial plateau was exposed, and the proximal osteotomy was performed taking lateral tibial plateau as reference. The lateral femoral osteotomy locator was installed after confirming the extension gap, and the testing membrane was installed after the anterior and posterior osteotomies were performed. The installation of knee joints was observed.

### 2.5. Observation Criteria

1. The knee joint scores (HSS): pain, function and activity, strength of joint motion, muscle strength, flexion deformity, and stability were utilized as assessment items. Before surgery (T_0_), 3 months after surgery (T_1_), 6 months after surgery (T_2_), 12 months after surgery (T_3_), and 24 months after surgery (T_4_) were the time nodes for comparison, and higher scores referred to better recovery of knee joints [12].
2. The Western Ontario and McMaster University (WOMAC) scores: WOMAC scores could completely evaluate the patients' joint pain, joint motion, and improvement of physical signs and lower WOMAC scores referred to better quality of life. Before surgery (T_0_), 3 months after surgery (T_1_), and 6 months after surgery (T_2_) were the time nodes for comparison [13].
3. The occurrence of complications: the complications included severe prosthesis fracture, secondary sepsis, patellar tendon rupture, pulmonary infection, periprosthetic infections, skin necrosis, neurovascular damage, and anterior knee pain, and the number of the cases was recorded.
4. The curative effect of TKA: if the physical signs of the affected knee joints basically disappeared and the WOMAC scores decreased by more than 95%, the patients were considered to be cured. If the physical signs of the affected knee joints were significantly improved and the WOMAC scores decreased by 70%–95%, it was considered significantly effective. If the physical signs of the affected knee joints were improved and the WOMAC scores decreased by 30%–70%, it was considered effective. If the criteria mentioned above were not met, it was considered ineffective. 3 months after surgery (T_1_) and 6 months after surgery (T_2_) were the time nodes for comparison [14, 15].

### 2.6. Statistic Treatment

In this study, the data processing software selected was SPSS 20.0 and the selected drawing software was GraphPad Prism 7 (GraphPad Software, San Diego, USA). This study included count data and measurement data and used the *X*^2^ test and *t* test. *P* < 0.05 indicated that the difference had a statistical significance.

## 3. Results

### 3.1. Analysis of the HSS Scores of Patients

**Figure 1:**
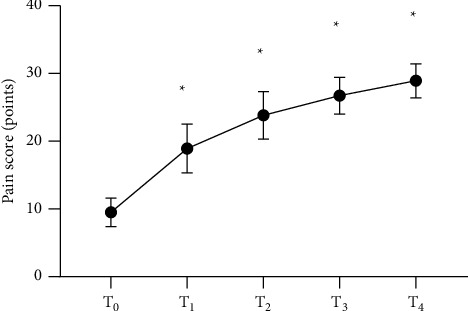
Analysis of pain scores of patients ($\bar{x}\pm s$, points). The horizontal axis from left to right represents before surgery (T_0_), 3 months after surgery (T_1_), 6 months after surgery (T_2_), 12 months after surgery (T_3_), and 24 months after surgery (T_4_), and the vertical axis represents the pain score (points). The pain scores at T_0_, T_1_, T_2_, T_3_, and T_4_ were (9.5 ± 2.1), (18.9 ± 3.6), (23.8 ± 3.5), (26.7 ± 2.7), and (28.9 ± 2.5), respectively. ^*∗*^Comparison of the time nodes after surgery with T0 (*P* < 0.001).

**Figure 2:**
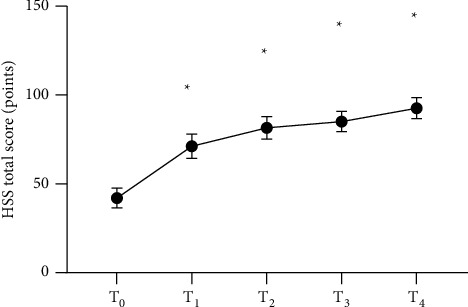
Analysis of total HSS scores of patients ($\bar{x}\pm s$, points). The horizontal axis of from left to right represents before surgery (T_0_), 3 months after surgery (T_1_), 6 months after surgery (T_2_), 12 months after surgery (T_3_), and 24 months after surgery (T_4_), and the vertical axis represents the total HSS score (points). The total HSS scores at T_0,_ T_1,_ T_2,_ T_3_, and T_4_ were (42.1 ± 5.6), (71.2 ± 6.9), (81.5 ± 6.3), (85.1 ± 5.7), and (92.6 ± 5.9), respectively. ^*∗*^Comparison of the time nodes after surgery with T_0_ (*P* < 0.05).

The HSS scores after surgery were significantly higher than the score before surgery (*P* < 0.05), and the pain scores of patients increased with time, which indicated that patients' pain was alleviated gradually, as shown in Figures 1 and 2 and Table 2.

### 3.2. Analysis of the WOMAC Scores of Patients

**Figure 3:**
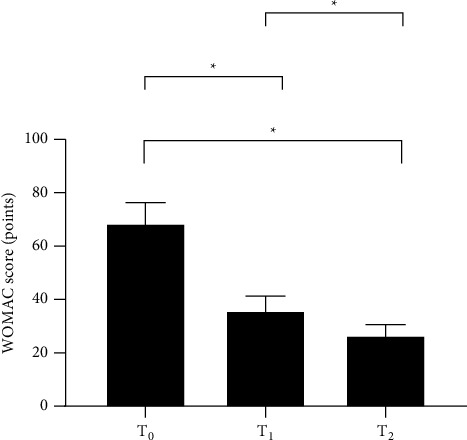
Analysis of WOMAC scores of patients ($\bar{x}\pm s$, points). The horizontal axis from left to right represents before surgery (T_0_), 3 months after surgery (T_1_), and 6 months after surgery (T_2_), and the vertical axis represents the WOMAC score (points). The WOMAC scores at T_0_, T_1_, and T_2_ were (68.5 ± 7.8), (35.9 ± 5.4), and (26.5 ± 4.1), respectively. ^*∗*^Comparison between T_0_ and T_1_, T_0_, and T_2_ and between T_1_ and T_2_ (*P* < 0.001).

The WOMAC scores after surgery were significantly lower than the score before surgery (*P* < 0.001), and the score at 6 months after surgery was significantly lower than that at 3 months after surgery, as shown in Figure 3.

### 3.3. Analysis of the Occurrence of Complications of Patients

**Figure 4:**
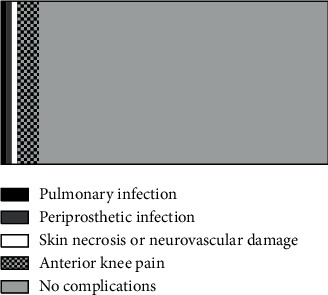
Analysis of occurrence of complications of patients. The black area represents the pulmonary infection (1 case), the dark grey area represents the periprosthetic infection (1 case), the white area represents the skin necrosis or neurovascular damage (1 case), the grid area represents the anterior knee pain (4 cases), and the light grey area represents no complications (53 cases).

The complications such as severe prosthesis fracture, secondary sepsis, and patellar tendon rupture were not found in patients, with the total incidence of complications of 11.7%, as shown in Figure 4.

### 3.4. The Evaluation of the Curative Effect of TKA of Patients

The effective rate of treatment of patients at 6 months after surgery (98.3%) was significantly higher than that at 3 months after surgery (*P* < 0.05), as given in Table 3.

## 4. Conclusion

This study concluded that WOMAC scores of patients after surgery were significantly lower than the score before surgery (*P* < 0.001), and the effective rate of treatment of patients at 6 months after surgery was 98.3%, indicating that TKA has a high value in improving the comprehensive condition of patients, accelerating their recovery after TKA, and significantly improving their quality of life. In the study of scholar Ackerman I.N, 121 cases with KOA who underwent TKA were followed up, and it was found that the WOMAC score at 3 months after surgery was (34.9 ± 5.2), while the score at 6 months after surgery was (26.4 ± 4.5), both of which were significantly lower than that before surgery (*P* < 0.001) [16–19], indicating that TKA can effectively relieve the symptoms and improve the quality of life of patients. It is noteworthy that this study only investigated the curative effect of patients within 6 months after surgery, and the long-term treatment outcomes cannot be judged comprehensively. It is generally believed in academic circles that the effective rate of treatment will decline at one year after TKA, but the decline is still higher than that at 3 months after surgery, indicating that the recovery of knee joint function tends to be stable at one year after surgery, and TKA has a stable treatment effect.

Furthermore, the complications such as severe prosthesis fracture, secondary sepsis, and patellar tendon rupture were not found in patients involved in this study, with the total incidence of complications of 11.7%, indicating that the safety of TKA is excellent. However, this study has a small sample quantity, and more samples are required in future studies for exploring the safety of TKA.

In conclusion, TKA can obviously improve the knee joint function and relieve pain of patients with KOA, with low incidence of complications and good quality of life of patients, which is worthy of further promotion and application.

## 5. Discussion

Pain and dysfunction in knee joints are the main clinical manifestations of patients with KOA, and the purpose of treatment should be relieving pain and improving the knee joint function, so as to improve the quality of life of patients [20–23]. This study showed that the HSS scores of patients with KOA after surgery were significantly higher than the score before surgery (*P* < 0.05), and the postoperative pain scores increased with time, indicating that the patients' pain was alleviated gradually. This is because TKA can reduce the release frequency of proinflammatory cytokines in patients, especially tumor necrosis factor (TNF-*α*) and interleukin-1*β* (IL-1*β*), which are closely related to the integrity of the articular cartilage tissue. These proinflammatory cytokines are important factors leading to KOA. TNF-*α* can increase secretion of matrix metalloproteinase-1, so as to speed up the release frequency of vascular endothelial growth factor and promote proliferation of endothelial cells, while IL-1*β* can further stimulate the activity of TNF-*α* and significantly increase secretion of the proinflammatory cytokines. TNF-*α* and IL-1*β* in combination can damage the knee joint function and enhance the sensitivity of pain receptor in patients. The pain receptor transmits the pain signal to sensory neuron till the sensory centre of cerebral cortex and makes patients feel more painful [24–27]. The study of scholar Christiansen M showed that TKA can reduce the concentration of TNF-*α* and IL-1*β* in patients, so as to reduce the inflammatory transmitter produced by chondrocytes, with less pain and better recovery effect of knee joints [28].

## Figures and Tables

**Table 1:** Comparison of general data of patients.

Gender	*N*	Age (years)		Disease duration (years)		Body mass index (kg/m^2^)		Lesion sites (*n*)		
		Range	Mean age	Range	Mean duration of disease	Range	Mean body mass index	Left	Right	Bilateral
Male	25	45–74	59.1 ± 3.2	6–12	6.2 ± 2.1	18–32	26.1 ± 2.1	5	10	10
Female	35	45–75	59.2 ± 3.1	6–11	6.3 ± 2.1	18–31	26.2 ± 2.3	8	10	17

**Table 2:** Analysis of HSS scores of patients ($\bar{x}\pm s$, points).

Time	Function and activity	Strength of joint motion	Muscle strength	Flexion deformity	Stability
T_0_	11.5 ± 2.2	8.2 ± 1.2	3.0 ± 1.4	1.3 ± 1.0	3.9 ± 1.2
T_1_	15.6 ± 3.1^*∗*^	10.5 ± 3.2^*∗*^	5.2 ± 1.6^*∗*^	5.9 ± 1.8^*∗*^	5.4 ± 1.8^*∗*^
T_2_	18.4 ± 2.8^*∗*^	14.6 ± 2.6^*∗*^	6.9 ± 2.4^*∗*^	7.2 ± 2.1^*∗*^	7.8 ± 2.1^*∗*^
T_3_	20.1 ± 3.6^*∗*^	16.0 ± 2.4^*∗*^	7.9 ± 1.8^*∗*^	8.4 ± 2.1^*∗*^	8.6 ± 1.6^*∗*^
T_4_	22.1 ± 2.0^*∗*^	17.1 ± 1.6^*∗*^	9.2 ± 2.0^*∗*^	9.3 ± 1.2^*∗*^	9.3 ± 0.9^*∗*^

^*∗*^Comparison of the time nodes after surgery with T_0_ (*P* < 0.05).

**Table 3:** Evaluation of the curative effect of TKA of patients.

Time	Cured	Significantly effective	Effective	Ineffective	Total effective cases
T_1_	3	24	26	7	53
T_2_	9	30	20	1	59
*X* ^2^	3.333	1.212	1.269	4.821	4.821
*P*	0.068	0.271	0.260	0.028	0.028

## Data Availability

The datasets used and/or analyzed during the current study are available from the corresponding author upon request.
